# Habitat Specialization and Airborne Dispersal Shape the Microbiome of a Gypsum Karst Cave

**DOI:** 10.1007/s00248-026-02745-y

**Published:** 2026-03-20

**Authors:** Tamara Martin-Pozas, Angel Fernandez-Cortes, Jose Maria Calaforra, Guillermo Ledesma-Hernandez, Soledad Cuezva, Sergio Sanchez-Moral, Cesareo Saiz-Jimenez, Valme Jurado

**Affiliations:** 1 Instituto de Recursos Naturales y Agrobiologia de Sevilla, IRNAS-CSIC, Sevilla, 41012 Spain; 2 Departamento de Biologia y Geologia, Universidad de Almeria, Almeria, Spain; 3 Museo Nacional de Ciencias Naturales, MNCN-CSIC, Madrid, Spain

**Keywords:** Gypsum caves, Biofilms, Sediments, Water pools, Air, *Crossiella*, wb1-P19, *Euzebyaceae*, Bioaerosols

## Abstract

The Gypsum Karst of Sorbas (Almeria, southeastern Spain) is a gypsiferous massif covering approximately 12 km² and constitutes one of the most extensively studied and internationally recognized gypsum karst systems. So far, only a limited number of studies have addressed microbial diversity in gypsum karst caves, and most of them have focused on a reduced number of habitats or specific cave compartments. Here, we characterize the prokaryotic diversity in biofilms, sediments, water and air in the gypsum cave of Covadura to compare the community composition across different habitats, assess connectivity among terrestrial, aquatic and aerial environments, examine associations with environmental and geochemical parameters, and provide baseline information for gypsum cave ecosystems. For this purpose, 28 samples were collected from different compartments distributed within the cave and above it, and analyzed using high-throughput 16 S rRNA gene sequencing. While dominant phyla, classes and genera resembled those of other karst systems, communities differed strongly among habitats. Water samples hosted distinct aquatic assemblages absent from biofilms, sediments, air and soil, indicating a clear separation between aquatic and non-aquatic cave systems. Biofilms were dominated by *Crossiella*, members of wb1-P19 and of the *Euzebyaceae*, which were also present in sediments, but scarce in water samples. Interestingly, *Crossiella* and wb1-P19 members were also detected in the cave air for the first time, suggesting their release from terrestrial communities and subsequent redistribution as bioaerosols and supporting cave air as a key dispersal vector that links stable terrestrial microbial reservoirs in Covadura Cave. This study reveals strong habitat specificity and high spatial heterogeneity in gypsum cave microbiomes, providing new ecological insights relevant for the conservation and management of cave ecosystems.

**Supplementary Information:**

The online version contains supplementary material available at 10.1007/s00248-026-02745-y.

## Introduction

In general, caves are poorly explored ecosystems compared to other terrestrial and aquatic environments at the surface. Most caves are considered extreme environments where life is limited due to severe environmental conditions (lack of light, alkaline pH, nutrient shortage etc.), however, caves are heavily colonized by microorganisms, which occupy all available habitats [1–4].

Current knowledge on microbial diversity and activity in caves, together with their distribution patterns and environmental controls, remains limited and is mainly conducted in show caves affected by conservation problems. In fact, the proliferation and expansion of specific microbial communities in cave habitats due to wrong management can lead to their irreversible deterioration [5–8].

Substrate composition, nutrient availability, and microclimatic factors are key determinants of microbial community structure in caves [9–11]. As such, gypsum caves are expected to harbor microbial communities that would differ significantly from those found in limestone systems. A recent study by Martin-Pozas et al. [12] in three Italian gypsum caves revealed distinct microbial assemblages between water and wall biofilms, but a surprising similarity in sediment communities across geographically separated sites.

Different studies on cave microbial communities have been reported, but on most cases focused in only one selected habitat, either wall rocks, ground sediments, speleothems, mineral deposits, biofilms and microbial mats, dripping water, pools, air, etc [13–16]. Less common are studies including two or three habitats, mostly weathered rock and sediments [2, 9, 17, 18].

However, studies comparing microbial communities in different cave habitats — or even among the most relevant ones, such as wall rocks, sediments, biofilms, water, and air — remain scarce [4, 12, 19–21]. As far as we know this is the first study where all cave habitats are investigated.

Previous studies showed significant differences in the composition of the bacterial community on different substrates suggesting physiological adaptations according to geochemical and environmental conditions [2, 4, 18, 21].

The caves of the Gypsum Karst of Sorbas, Southeast Spain, and their microclimatology, hydrogeology, geomorphology etc., have been widely studied [22–26]. However, the geomicrobiology of the Gypsum Karst of Sorbas has been only the subject of very recent research [16, 27, 28].

The aim of this study was to characterized the prokaryotic diversity across multiple habitats (air, water, soil, sediments and biofilms) within Covadura Cave, a representative and ecologically important site located in the Gypsum Karst of Sorbas (Almeria, Spain), using high-throughput 16 S rRNA gene sequencing. The questions to be addressed in this study are (i) how bacteria are transferred from the exterior to the subterranean environment, (ii) whether, how, and where this transport occurs, and (iii) what are the dispersal mechanisms and potential reservoirs that lead to the presence of a major bacterial core in the sediments and biofilms within this cave system.

Additionally, we compared community composition across the different environments to assess connectivity among terrestrial, aquatic and aerial environments, and examined the relationship between microbial communities, local geochemical features and local climatic ventilation conditions, in order to provide baseline data for future conservation and management strategies for gypsum cave ecosystems.

## Materials and Methods

### Geological and Climate Settings

The Gypsum Karst of Sorbas (Almeria, southeastern Spain) has developed within a Messinian gypsiferous massif, covering an area of 12 km^2^, comprising 1000 sinkholes and caves and more than 100 km of cave passages [29]. It forms a multilayer karst aquifer developed in interbedded gypsum and marls, whose geological evolution and geomorphology have been described in detail elsewhere [22, 23]. This constitutes an exhaustive sampling; nevertheless, by conducting sampling during the same season, ideally within a single field campaign, would minimize potential seasonal variability.

**Fig. 1:**
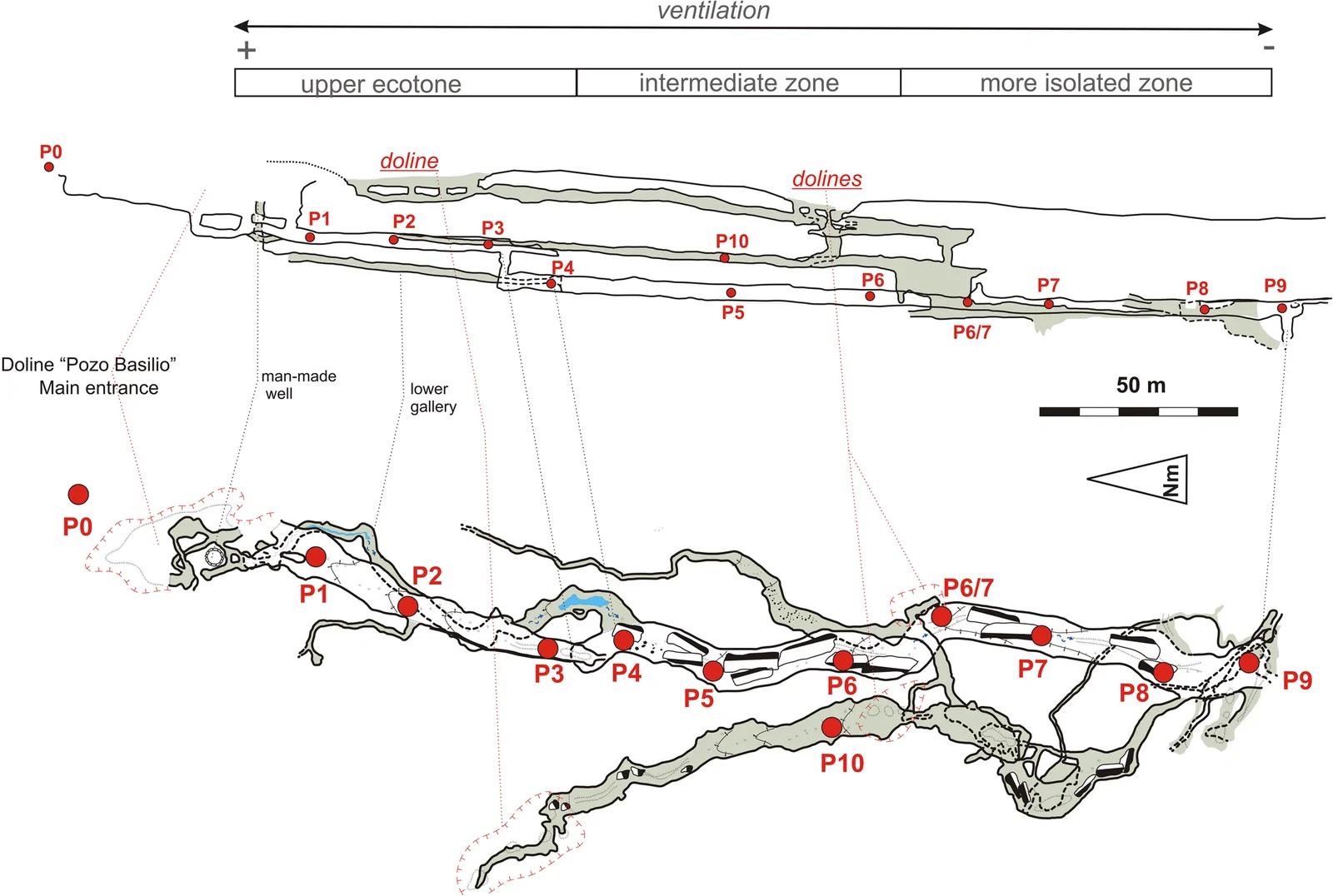
Location of microclimatic and microbiological sampling points (P1–P10) distributed along the main galleries of Covadura Cave. The cave galleries are grouped into three sectors according to their aerodynamic connection with the exterior and their microclimate stability. Cave maps (floor plans and profiles) modified from Ayuso et al. [32]

Covadura Cave, located on the northwest side of the karst (UTM ETRS89 30 S: 582803–4108926, altitude: 405.9 m a.s.l.), is one of the longest and deepest gypsum caves in Spain. It consists of six different levels of interstratified galleries, along a depth of 126 m and a total length of 4.25 km. The galleries sampled in this study have a length of approximately 275 m along two interstratified marls layers, with a width of 4–5 m and a height of 2–3 m on average (Fig. 1).

Covadura Cave is home to a significant population of bats and access is currently restricted during the hibernation period (October-March). Occasional activation of the watercourse during extreme rainfall events has been reported, leading to partial flooding that affected mainly the cave floor and sediment deposits, while most of the biofilms in walls and ceilings of the cave remain unaffected [25, 30].

The region, one of the most arid in Europe, is characterized by a semi-arid Mediterranean climate, with an average annual temperature of around 19.5 °C, a low annual rainfall of about 210 mm and sparse vegetation cover dominated by shrubs and biocrusts [24].

### Sampling

During November 2010, June 2022 and October 2023, a total of 28 samples were collected from different environments in Covadura Cave: cave biofilms (*n* = 11), cave sediments (*n* = 5), cave water pools (*n* = 4), cave air (*n* = 4), exterior air (*n* = 2) and soil above the cave (*n* = 2). Detailed information on sample locations is provided in Fig. 1 and Table S1.

Air samples were obtained using a Coriolis µ air sampler (Bertin Technologies, France) that draws air through a liquid medium (0.005% Triton X) designed to capture bioaerosols [31]. A total of 6 m^3^ of air was sampled at 300 L/min for 20 min and then filtered through 0.22 μm membrane filters.

Approximately 500 mg of sediments and soil and 150 mg of biofilms from walls and ceilings were collected using a sterile scalpel and transferred to 1.5 ml Eppendorf tubes. Additional samples (~ 50 g of sediment and soil) were collected in sterile bags for physicochemical analyses.

Water samples (1 L) were collected in sterile bottles and filtered using a vacuum system through 0.22 μm membrane filters. Filtered water was also used for the assessment of physico-chemical parameters.

All samples, membrane filters, sediments and biofilms, used for molecular analyses, were transported on dry ice to the laboratory, where they were immediately processed or stored at − 80 °C until further analysis. Samples for microscopy and for analysis of physico-chemical parameters were stored at 4 °C until processing. Water samples were processed within 24 h of sampling and sediment samples were oven-dried at 40 °C within 48 h of sampling.

### Environmental and Physicochemical Characterization

Cave microclimatic parameters (air temperature, relative humidity, gaseous composition) physicochemical properties of sediments and waters were measured to characterize the environmental context of the samples. Biofilm morphology was examined by stereomicroscopy and environmental scanning electron microscopy to support biofilm classification. Details are provided in the Supplementary Information.

### DNA Extraction and 16 S rRNA Gene Amplicon Sequencing

DNA was extracted from all samples using the FastDNA SPIN Kit for Soil (MP Biomedicals, Illkrich, France), following the manufacturer’s protocol. Library preparation and high-throughput sequencing were carried out at FISABIO Institute (Valencia, Spain). The V3-V4 regions of the 16S small subunit ribosomal RNA (SSU rRNA) gene were amplified using primer sequences 341F (5’-CCTACGGGNGGCWGCAG-3’) and 805R (5’-GACTACHVGGGTATCTAATCC-3’) [33]. Negative controls without DNA were included during both the extraction and library preparation processes to monitor possible contamination. Amplicons were sequenced using Illumina MiSeq and 2 × 300 paired-end.

### Data Analyses

The raw data was processed using QIIME 2 [34] with DADA2 [35]. Taxonomic assignment was determined using the q2-feature-classifier plugin with a Naive Bayesian classifier pre-trained on the SILVA 138 database for the V3-V4 region [36]. Sequences identified as chloroplast and mitochondria were removed.

Data from QIIME 2 were imported to R (version 4.3.1) and processed using the *phyloseq* package [37]. Alpha diversity indices (Chao1, Shannon, and Simpson) were calculated with the *vegan* package. Rarefaction curves were calculated to assess sequencing depth. Differences in alpha diversity between different environments were evaluated using the Kruskal-Wallis test followed by Dunn’s post hoc comparisons. The results for environments with fewer than five samples were interpreted with caution due to the limited statistical power.

Beta diversity was assessed using Bray–Curtis dissimilarity and visualized by non-metric multidimensional scaling (NMDS) with the metaMDS function in *vegan*. Differences in community composition in different environments were assessed using analysis of similarity (ANOSIM) and permutational multivariate analysis of variance (PERMANOVA). Shared and exclusive genera among cave habitats were explored using Venn diagrams.

The core microbiome of each habitat was defined at the genus level as taxa with a relative abundance > 0.01% and present in at least 50% of the samples within a group. Associations between genera and habitats were assessed using the indicator value (IndVal) index implemented in the *indicspecies* package. Co-occurrence network analyses were performed for sample groups with *n* ≥ 5. ASVs were agglomerated at the genus level and filtered by prevalence (present in ≥ 40% of samples within the group) and mean relative abundance (≥ 0.001). Pairwise associations were then calculated using Spearman’s rank correlations (Hmisc rcorr). Only strong and significant correlations were retained (|ρ| ≥ 0.6 and *p* < 0.05).

## Results

### Microenvironmental Zoning

**Fig. 2:**
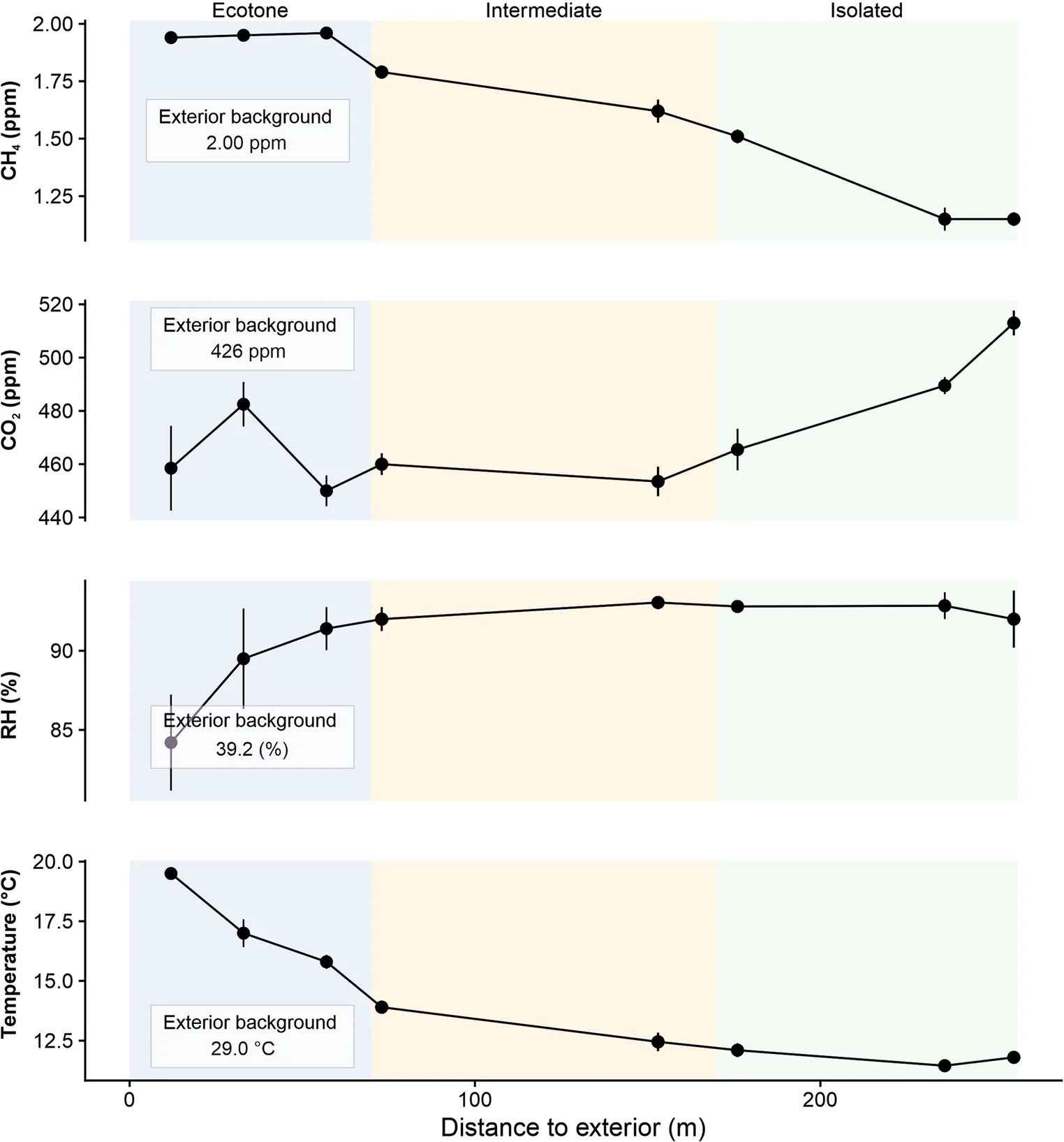
Microenvironmental zoning. Profiles of CH₄, CO₂, relative humidity (RH) and temperature plotted against distance from the cave entrance. Colored backgrounds indicate the three cave sectors defined in this study (ecotone, intermediate and isolated). Points represent the mean values measured at the sampling points

Covadura is a well-ventilated cave, with mean CO₂ values close to atmospheric levels (464 ppm), with lower temperatures (11.8–17.4 °C) and higher relative humidity (> 88%) than the exterior (Tables S2). A slight environmental gradient from the entrance toward the inner cave sections was characterized by a progressive decrease in temperature and CH₄ concentration. Based on these results, three sectors of galleries were identified (Figs. 1 and 2): (i) the upper ecotone sector (P1 to P3), characterized by higher temperature and substantial air renewal; (ii) an intermediate sector (P4 to P6) with moderate air exchange; and (iii) the innermost, less ventilated and coldest sector (P6/7 to P9).

### Chemistry of Karst Water and Cave Sediments

Cave waters showed a homogeneous calcium sulphate–bicarbonate composition, slightly alkaline pH (pH 7.5) and had low organic matter content (Table S3). Nitrate concentrations were generally low, but increased in the innermost water sample (P9-W). Cave sediments were slightly alkaline (pH 7.6–7.8) and presented low organic matter content with differences in nitrogen, phosphorous and mayor cation content (Table S4). An exception was sample P4-S, which exhibited higher organic matter content, more similar to the exterior soil.

### Microscopy

Diverse biofilm morphologies were observed in Covadura Cave, with different types of biofilms occasionally growing in close proximity. Based on macroscopic and microscopic features, the biofilms were classified into two types—yellow and white— based on color, elevation, margin and cell morphology (Table S5). All biofilms showed a filamentous shape, irregular margins and variable sizes ranging from punctiform (< 0.5 mm) to large areas (> 1 mm), without evidence of biomineralization.

Yellow biofilms showed homogeneous morphologies dominated by long bacillary filaments with a fluffy appearance and smooth cocci (Figs. S1 and S3). In contrast, white biofilms exhibited two predominant morphologies: long bacillary filaments with ornamented surfaces and long smooth filaments (Figs. S2 and S4).

### Microbial Diversity

The dataset comprised 28 samples, with a total of 2,202,610 high-quality reads, clustered into 32,660 ASVs. Rarefaction curves showed that the sequencing depth was sufficient for most samples (Fig. S5).

**Fig. 3:**
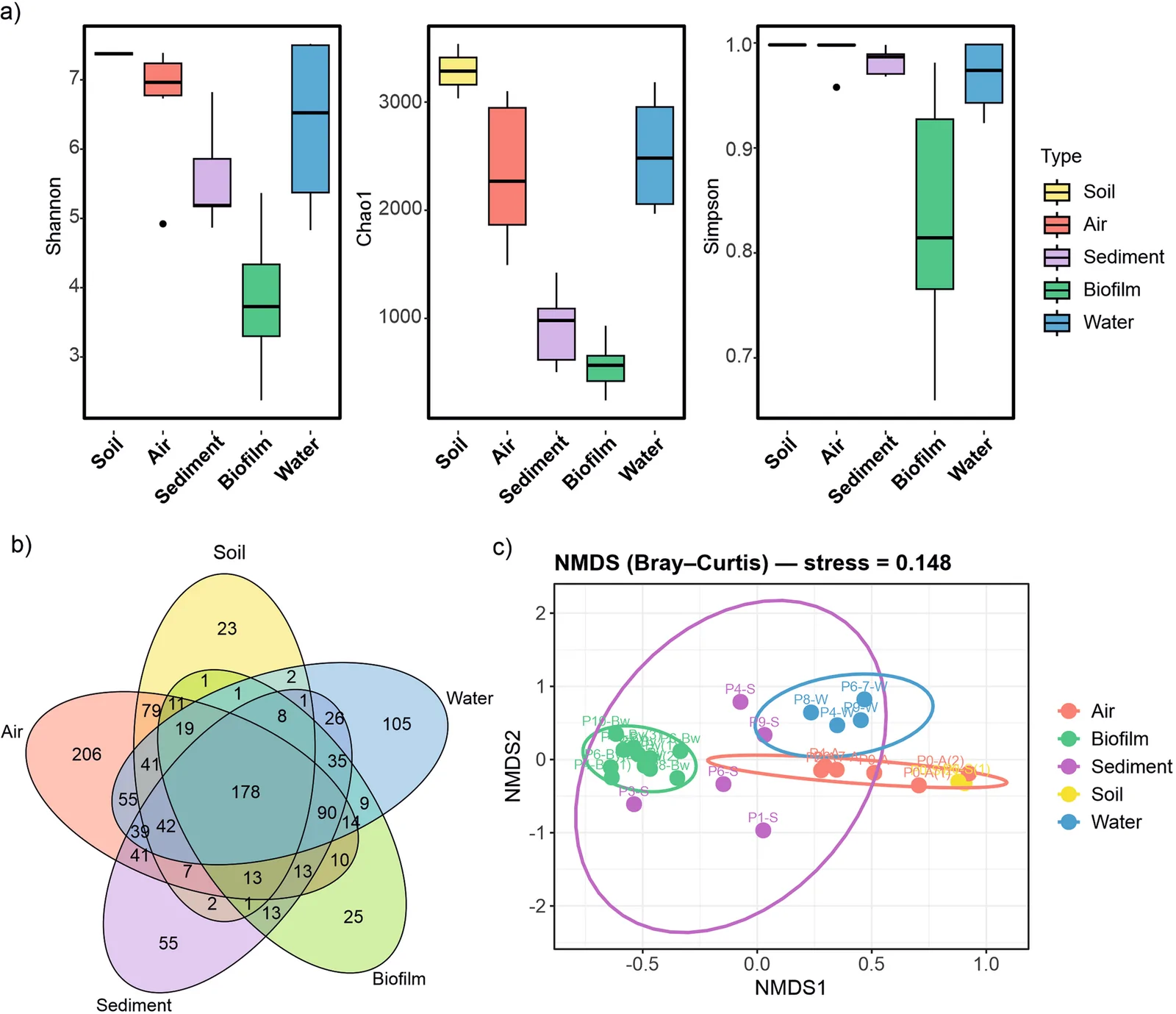
**a** Alpha diversity indices (Shannon, Chao1, and Simpson). **b** Venn diagram representation of shared and unique prokaryotic genera across different cave environments. **c** Non-metric multidimensional scaling (NMDS) ordination based on Bray–Curtis dissimilarity of microbial communities in different habitats of Covadura Cave

Alpha diversity indices showed that biofilms exhibited lower microbial richness and diversity compared to other habitats, being significantly less diverse than air and water samples (Fig. 3a; Table S6). Within the air group, sample P4-A appeared as an outlier with lower Shannon (4.94) and Simpson (0.96) values than the other air samples, which had higher diversity values (Shannon > 7, Simpson = 1). Shared and exclusive genera across habitats are summarized in the Venn diagram (Fig. 2b), with a total of 1,165 genera identified. Of these, 178 genera were shared by all five habitats, while the highest number of unique genera was observed in air samples (217) (Fig. 3b).

The analysis of beta diversity in the genus sample revealed that the habitat type influenced the bacterial community. NMDS ordination based on Bray-Curtis dissimilarity showed three independent clusters: air, biofilm and water samples, while cave sediments exhibited a more dispersed distribution (Fig. 3c). ANOSIM and PERMANOVA results confirmed these patterns, and habitat type explained approximately 27% of the total compositional variance (*p* < 0.05; Table S7).

Although white and yellow biofilms were analyzed together due to their spatial co-occurrence, beta diversity analyses revealed weak but statistically significant compositional differentiation within the biofilm group (ANOSIM *R* = 0.30, *p* = 0.03). No significant differences were observed between biofilm samples collected in 2010 and 2022.

### Taxonomic Composition

All samples contained negligible percentages of *Archaea*, in contrast to 99.72%–100% of *Bacteria*, except for the sediment sample P4-S, which showed 96.58% of *Bacteria* and 3.42% of *Archaea* (*Nanoarchaeota*).

**Fig. 4:**
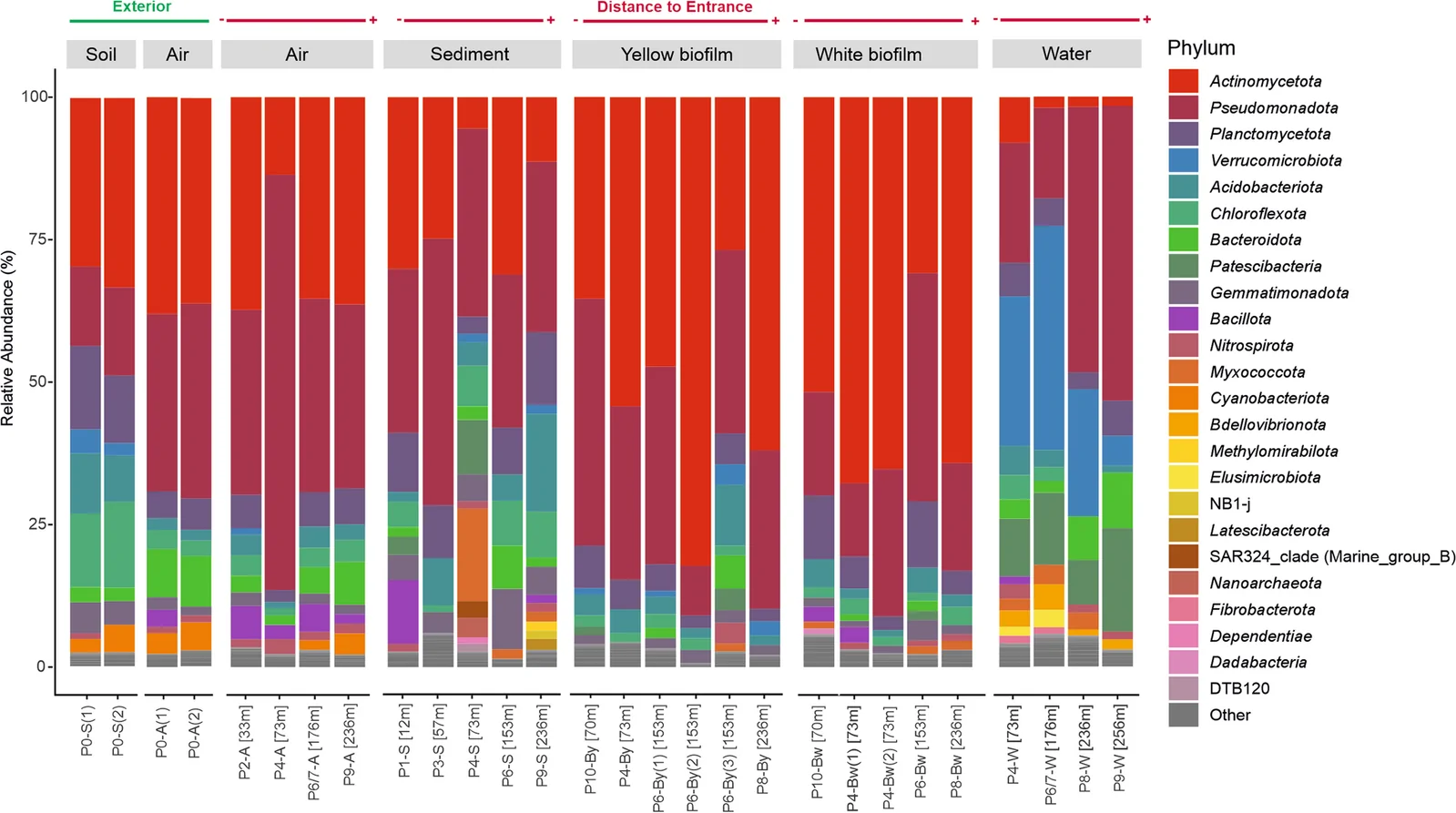
Phylum-level composition of microbial communities. Bar plot represented the relative abundance (%) of bacterial and archaeal phyla in exterior and cave samples. The samples are ordered by habitat type and increasing distance from the cave entrance. Phyla with relative abundances below 1% in all samples were grouped as “Other”. Label codes P# correspond to the sampling point of Fig. 1, each one with the following distance (in meters) to the main cave entrance (P0, exterior): 12 m (P1), 33 m (P2), 57 m (P3), 70 m (P10), 73 m (P4), 118 m (P5), 153 m (P6), 176 m (P6/7), 236 m (P8) and 256 m (P9)

The two most abundant phyla in all samples were *Actinomycetota* and *Pseudomonadota*, although their relative abundance varied among sample types (Fig. 4). *Actinomycetota* was particularly abundant in biofilms (26.82%–82.21%). Other phyla present in all samples but with variable abundances among sample types were *Acidobacteriota* (0.76%–17.14%), *Planctomycetota* (2.06%–14.68%), *Chloroflexota* (0.32%–12.91%) and *Bacteroidota* (0%–9.76%). *Candidatus* (*Ca.*) Patescibacteria (7.83%–18.17%) and *Verrucomicrobiota* (5.21%–39.44%) were abundant in water samples, while they remain negligible or very low in most other habitats. Exceptions were observed in the sediment sample P4-S (*Ca.* Patescibacteria, 9.58%) and in the exterior soil sample P0-S (*Verrucomicrobiota*, 4.20%).

In addition to the dominant phyla, *Planctomycetota* was relatively more abundant in biofilms, sediments and soil, while *Chloroflexota* was generally more abundant in sediments and soil. *Elusimicrobiota*,* Fibrobacterota* and *Bdellovibrionota* were almost exclusive of the water samples.

**Fig. 5:**
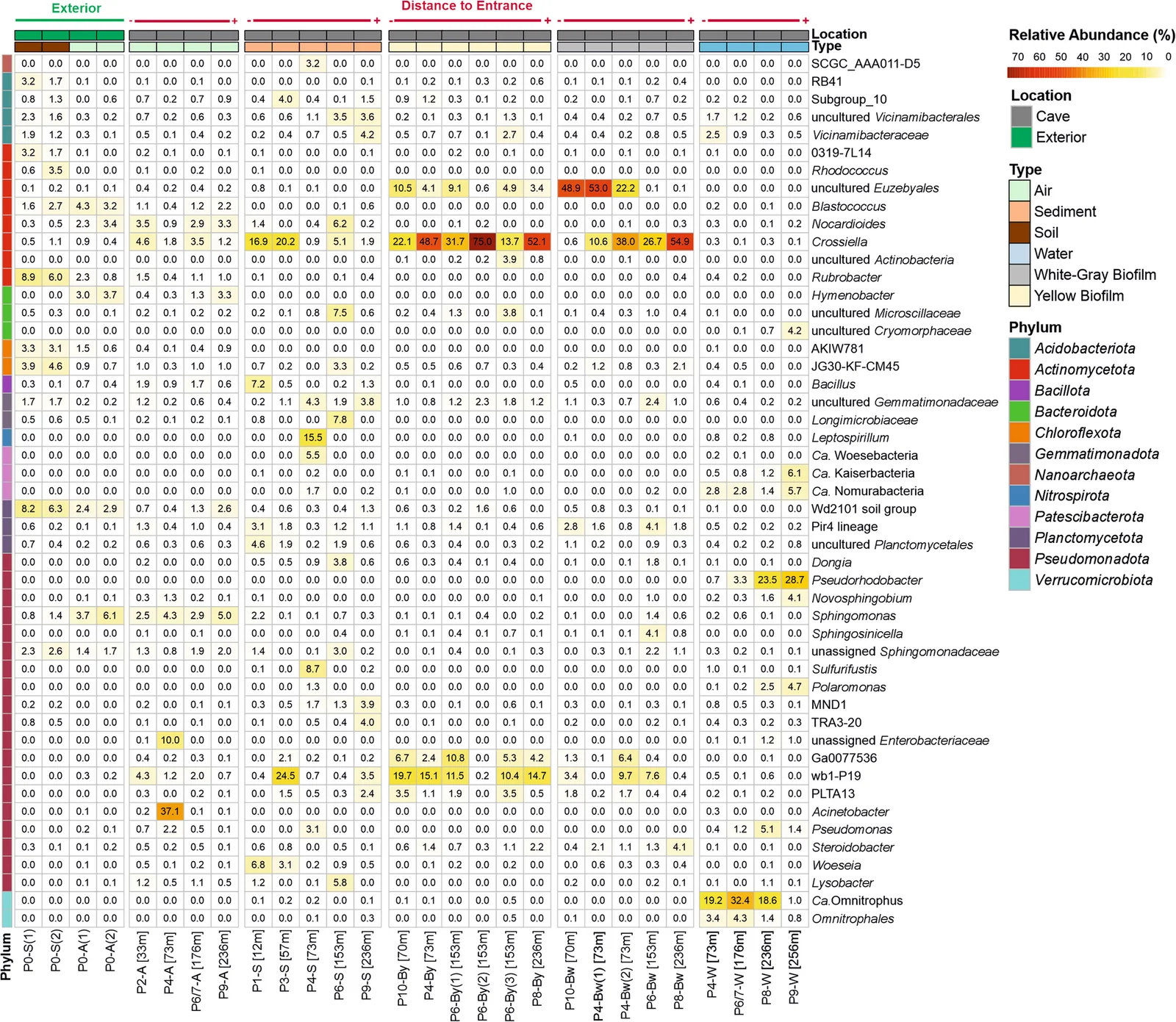
Heatmap showing the most abundant genera with relative abundance > 3% in Covadura in at least one sample. Samples are grouped by habitat type (air, sediment, soil, water, white biofilm, and yellow biofilm) and ordered by increasing distance from the cave entrance. Label codes P# correspond to the sampling point of Fig. 1, each one with the following distance (in meters) to the main cave entrance (P0, exterior): 12 m (P1), 33 m (P2), 57 m (P3), 70 m (P10), 73 m (P4), 118 m (P5), 153 m (P6), 176 m (P6/7), 236 m (P8) and 256 m (P9)

**Fig. 6:**
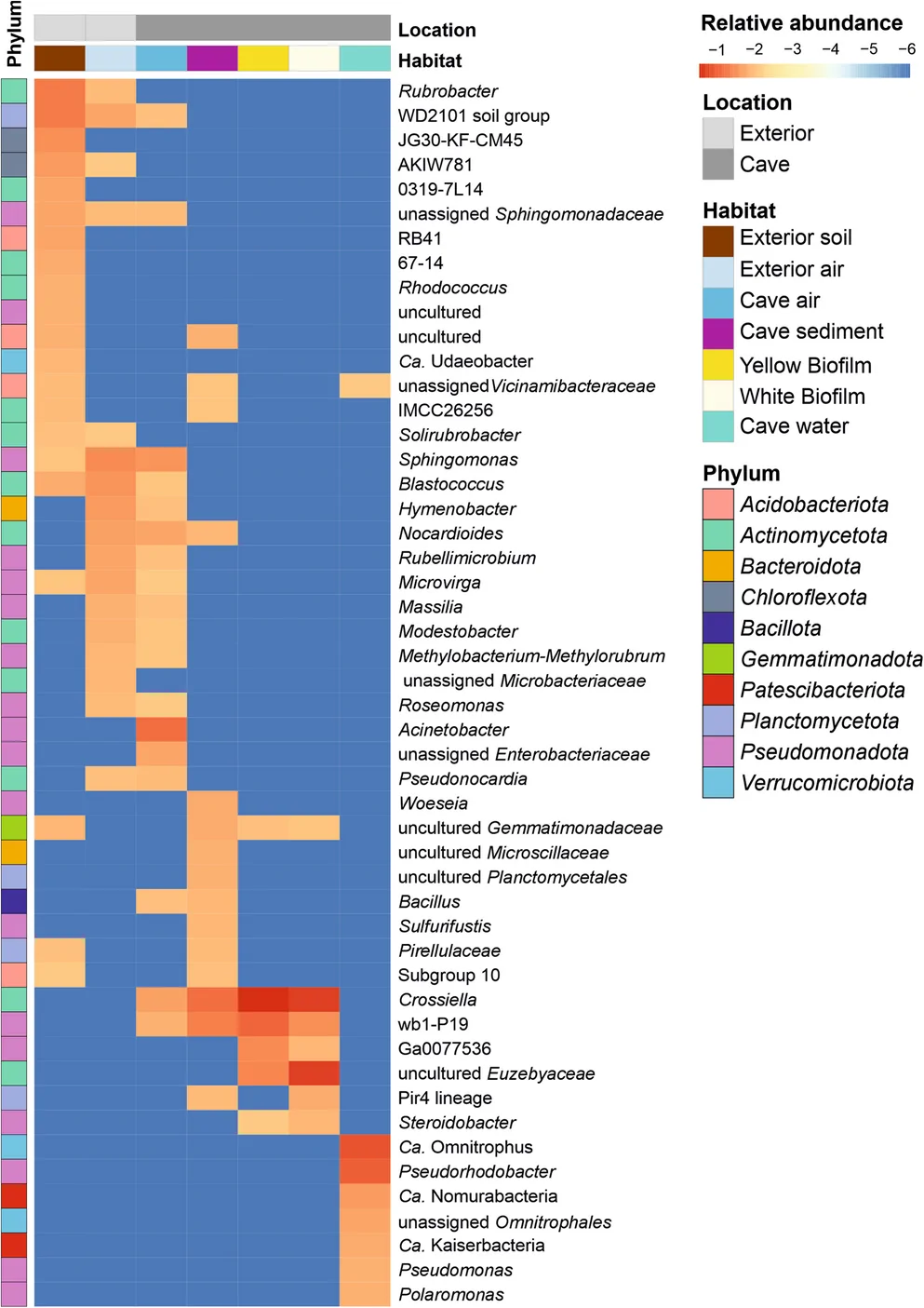
Core microbiome heatmap. Heatmap of log₁₀-transformed mean relative abundance of core microbial taxa in different habitats. Only 50 most relevant taxa classified as core members in at least one group are shown. The core microbiome was defined as genera with a mean relative abundance ≥ 0.5% and present in ≥ 50% of samples within a habitat (*n* ≥ 2). Warmer colors (− 1 to − 3) indicate high average relative abundance and cooler colors (− 4 to − 6) indicate low abundance

At the genus level, the composition of the microbial community was dominated by a small number of taxa (Figs. 5 and 6). In total, 305 genera showed relative abundances > 1% in at least one sample. Among them, the occurrence and high relative abundances of three genera, *Crossiella*, an uncultured wb1-P19 and *Euzebyaceae*, is notable in most of the samples. In the biofilms, *Crossiella* reached the highest abundances (0.65%–75.03%), followed by uncultured *Euzebyaceae* (0.09%–53.01%) and wb1-P19 (0%–19.66%). However, uncultured *Euzebyaceae* showed a more restricted distribution, being detected in all air and soil samples, but in most sediment and water samples with very low abundance (0.01%–0.80%). The abundances of Ga0077536 (0.08%–10.79%) and *Steroidobacter* (0.31%–4.15%) were also notable in both biofilm types.

Sediment samples showed a high variability of abundant genera (> 5%), some of them broadly distributed and others highly localized. *Crossiella* (0.87%–20.25%) and wb1-P19 (0.41%–24.50%) were also significant in sediments. Other genera were restricted to one or two sediment samples. *Leptospirillum* and *Sulfurifustis* reached high abundances in sample P4-S (15.47% and 8.73%, respectively) but remained negligible in all other sediments and habitats. *Woeseia* and uncultured *Microscillaceae* appeared broadly distributed within the samples but with high relative abundances in specific sediment samples. *Woeseia* showed highest relative abundances in ecotone sediments (6.83% in P1-S and 3.07% in P3-S), while uncultured *Microscillaceae* presented high relative abundances in P6-S (7.51%) and secondary increases in some P6-associated biofilms. *Nocardioides* and *Lysobacter* were present in individual sediment samples with high abundances (up to 6.21% and 5.77% in P6-S, respectively). *Bacillus* was widespread but showed a high relative abundance in the P1-S sediment sample (P7.17%). These genera were also identified as significant indicators in soil, air, water and sediment samples, supporting their recurrence but also habitat-dependent occurrence (Fig. S6).

The water samples displayed a completely different spectrum. *Ca.* Omnitrophus was the most abundant genus in cave waters (1.05%–32.44%), followed by *Pseudorhodobacter* (0.74%–28.68%); both taxa were negligible in all other habitats. Other well represented taxa in cave water were *Ca.* Kaiserbacteria (0.52–6.14%), *Ca.* Nomurabacteria (1.37–5.67%), *Polaromonas* (0.06–4.71%) and uncultured members of *Omnitrophales* (0.83–4.31%) and *Parcubacteria* (0.74–1.61%). IndVal analyses confirmed *Polaromonas*, unassigned *Parcubacteria* and the order *Omnitrophales* as water indicators (Fig. S6). Although *Pseudomonas* reached high abundances in water samples (0.44–5.14%), this genus was detected in different habitats, generally at low relative abundances.

The cave air samples showed high relative abundances of *Crossiella* (1.24%–4.64%) and wb1-P19 (0.74%–4.33%), while both were present at very low abundances in the exterior air. Other genera with high relative abundances in cave air samples were *Acinetobacter* (37.08%), *Sphingomonas* (5.04%) and unassigned *Enterobacteriaceae* (10.02%). These taxa, reached high relative abundances in only one cave air sample. However, *Microvirga* and *Blastococcus* were detected in all air and sediment samples, but at generally low abundances. A similar pattern was observed in *Sphingomonas*, which was present in all samples.

The soil above the cave presented high relative abundances of *Rubrobacter* (6.00–8.89%) and WD2101_soil_group (6.26–8.23%), while it remained at lower abundances in cave habitats.

### Co-Occurrence Network Analysis

Co-occurrence networks were constructed separately for white biofilms, yellow biofilms and cave sediments to study the potential associations between dominant genera (Figs. S7–S9). In general, the networks were modular and dominated by positive correlations suggesting prevalent co-occurrence patterns within each habitat. Cave sediments showed a denser network (134 taxa connected by 141 positive and 74 negative correlations) than yellow biofilms (93 taxa connected by 120 positive and 58 negative associations) and white biofilms (95 taxa connected by 72 positive and 36 negative associations). Cave sediment networks showed higher node connectivity and a more clustered structure indicative of greater ecological heterogeneity and potential habitat-specific structuring. In contrast, biofilm networks showed lower node degrees and a more compact structure, consistent with more specialized and compositionally stable communities. In cave sediments, *Nocardioides*, *Woeseia* and *Crossiella* appeared as highly prevalent taxa participating in a dense co-occurrence module, suggesting their central role in structuring sediment-associated microbial assemblages.

## Discussion

### Habitat Determines the Microbial Community Structure in Covadura Cave

Microbial communities revealed differentiation in diverse habitats and ecosystems [38–40]. However, habitat differentiation in caves remains relatively unexplored, since most of this evidence is based on a limited number of compartments, typically sediments and weathered rocks [17, 18]. Some studies in volcanic, limestone and gypsum caves have shown that microbial communities cluster primarily according to habitat type (air, sediment, biofilms or water) [12, 20, 41, 42].

In this context, our results confirm that the Covadura Cave microbiome does not form a continuum, but is instead structured into distinct habitats, with limited connectivity between aquatic and terrestrial environments. This was exemplified in the Venn diagram which showed a higher proportion of habitat-specific than shared genera. Excluding the exterior samples, 10% of genera were shared by air, water, biofilm and sediment samples, suggesting a limited core of recurrent cave-associated taxa. Notably, most shared genera were unevenly distributed across habitats, reinforcing the idea of strong habitat specialization rather than a homogeneous cave-wide microbiome. Beta-diversity analyses further indicated a clear separation among air, biofilm and water communities, while sediments showed a more dispersed pattern with partial overlap with biofilms, consistent with shared substrate-associated taxa and stronger local variability in sediments (Fig. 3c).

Microenvironmental zonation does not appear to be a major determinant of whole-community structure, but some specific taxa displayed consistent distributional trends along the entrance–interior section in sediment and water. For example, *Crossiella* reached its highest relative abundances in ecotonal sediments (P1-S and P3-S) and showed a gradual tendency to decrease toward the inner cave sediments.

In the aquatic habitats, *Pseudorhodobacter* and other groundwater-associated genera (e.g. *Ca.* Kaiserbacteria and *Polaromonas*) increased markedly toward the inner cave waters, reaching high relative abundances at isolated sites, which coincided with localized increases in organic carbon and nitrate concentrations. In contrast, typical soil- and dust-associated taxa such as *Rubrobacter* were abundant in exterior soils and air but became rare or absent in the inner cave habitats of Covadura [43–46]. This pattern suggests that the presence of *Rubrobacter* in Covadura is mainly driven by external environmental inputs, in agreement with recent global soil and cave studies [47, 48]. In Covadura Cave, *Rubrobacter* was associated with air, water and exterior soil (Fig. S6) while other cave studies have reported this genus as an indicator of rock-associated communities. However, its limited persistence in inner cave substrates suggests passive transport rather than successful establishment within the subterranean environment.

Overall, habitat type explained a substantial fraction of the compositional variance, whereas microenvironmental zonation did not significantly influence whole-community composition, indicating that substrate properties exert greater control than spatial position in Covadura Cave.

### Biofilms as the Most Stable and Compositionally Homogeneous Subterranean Community

Biofilms represent the most stable associations of microorganisms in Covadura Cave with comparatively consistent composition in different microenvironmental zones.

The high abundances of the genus *Crossiella* in the biofilms and sediments of Covadura contrasted with its low abundances in the water and soil samples. The ecology and distribution of *Crossiella* has recently been reviewed, highlighting its dominance in cave systems worldwide where it has been linked to processes such as carbon sequestration, biomineralization and antimicrobial production [49–51]. Consistent with our results, many authors have reported its particular abundance in cave biofilms [12, 14, 16, 52]. Beyond caves, *Crossiella* has also been reported in diverse environments (e.g., soils, rhizosphere, building stones, mines, freshwaters and marine sediments) that cover a wide geographical distribution on different continents, including Antarctica [51].

The genus wb1-P19 showed a distribution pattern similar to *Crossiella*, with high relative abundances in biofilms and sediments and low abundances in waters and soils. Many authors reported high relative abundances of wb1-P19 in caves worldwide distributed [4, 12, 14, 16] and frequently reported in soils [53, 54]. Recent work has recovered nearly complete MAGs of wb1-P19 from caves and suggested a role in methane consumption [55], consistent with its recurrence in cave habitats and with caves that function as active methane sinks [56].

The high abundance of uncultured *Euzebyaceae* in biofilms is notable, with insignificant relative abundances in waters, soil, sediments and air samples from Covadura Cave. Members of this family have been reported in extreme environments and habitats with high salt concentrations, such as saline and alkaline soils, salt mines, and sediments of desiccated salt lakes [57]. Although *Euzebyaceae* is the second most abundant actinobacterial phylotype in wall-attached biofilms worldwide, its specific ecological roles and metabolic functionalities remain largely elusive [14, 16, 52, 58, 59].

The genera Ga0077536 and *Steroidobacter* were recurrent in Covadura biofilms while remaining insignificant in other habitats, a pattern also reported in other caves [14, 16, 18, 42]. These taxa are typically detected at low abundances in the subsurface but can become highly dominant in moonmilk deposits [60, 61]. Although their specific ecological roles within subsurface ecosystems are still poorly understood, Ga0077536 was predicted to possess chemoorganotrophic or methylotrophic properties [60]. On the other hand, *Steroidobacter* is a chemoheterotrophic, usually soil-associated genus [62, 63].

These results indicate that, despite their visual and compositional differences, Covadura biofilms share a conserved actinobacterial core dominated by *Crossiella* and *Euzebyaceae*. This recurrent core, coupled with their relatively low compositional variability across microenvironmental zones, reinforces biofilms as the most stable and compositionally homogeneous microbial community within the cave.

### Sediments Presented a High Microbial Heterogeneity

Sediment communities were dominated by members of *Pseudomonadota*,* Actinomycetota*,* Planctomycetota*,* Gemmatimonadota*,* Chloroflexota* and *Bacteroidota*, and the classes *Alphaproteobacteria*,* Gammaproteobacteria*, and *Actinobacteria*, with relative abundances above 10% in most samples. Similar abundances but only in one sample were obtained for *Vicinamibacteria* and *Bacilli*. These abundances of phyla and classes are commonly found in other cave sediments [18, 20] and topsoils [3, 11, 45].

In contrast to biofilms, sediment prokaryotic communities were highly heterogeneous within Covadura Cave (Fig. 3c), suggesting stronger influence of local environmental and physicochemical conditions than other cave habitats. A clear example is the marl sediment P4-S, which showed relatively high abundances of *Leptospirillum*, *Sulfurifustis*, *Ca.* Woesebacteria (syn. *Ca*. Microgenomatia) and *Nanoarchaeia*, taxa largely absent from other cave samples, consistent with a distinct local geochemical context. Sample P4-S also exhibited relatively high total organic carbon content (1.15%), probably related to recurrent organic matter inputs driven by water condensation and a direct connection to the exterior through an upper parallel gallery, where stagnant waters may accumulate dung falling through the well at the P4 site [28].

The presence of *Nanoarchaeia* (SCGC_AAA011-D5), *Leptospirillum*, *Ca.* Microgenomatia and *Sulfurifustis* have been previously reported in groundwater systems [64–66], marl sediments from cave pools [13] and deep subsurface aquifers [67, 68]. Genomic studies indicate that *Sulfurifustis* and *Leptospirillum* are involved in inorganic carbon fixation under suboxic conditions, acting as sulfur- and iron-oxidizers, respectively [69, 70], while *Ca.* Woesebacteria are consistent with microoxic environments where they may act as symbionts or fermenters with simplified metabolisms [71].

Of particular interest was the recurrent detection of *Crossiella* and wb1-P19 in sediment samples, where they reached high relative abundances, especially in those from the ecotone zone, comparable to those observed in biofilms. A similar pattern was reported in another Spanish karstic cave (Pindal Cave), where biofilms, water and sediments were compared across different locations within the same cave [42]. Several studies have proposed *Crossiella* and wb1-P19 as members of the core microbiome of cave biofilms [10, 12, 42], with *Crossiella* identified as an indicator taxon of cave rocks and wb1-P19 suggested as a keystone member in limestone cave communities [20]. Together, these observations suggest that sediments and biofilms share part of their bacterial communities, and that both taxa show a clear preference for terrestrial cave habitats such as wall biofilms, rocks and sediments.

### Cave Waters Host a Different Microbiome

The comparison between water and other cave habitats revealed marked differences in microbial composition, indicating clear specificity for cave waters. Covadura waters showed a distinct microbial profile characterized by the high relative abundance of *Verrucomicrobiota* and *Ca.* Patescibacteria, and to a lesser extent *Bdellovibrionota*, *Elusimicrobiota* and *Fibrobacterota*, phyla commonly associated with aquatic environments [72–74] and largely absent or present at very low abundances in other cave habitats.

Water communities were dominated by a limited number of groundwater-associated taxa, including *Ca.* Omnitrophus, unassigned *Parcubacteria*, *Pseudorhodobacter*, *Polaromonas*, and members of *Ca.* Kaiserbacteria and *Ca.* Nomurabacteria, which were rare or absent in sediments, biofilms, soils and air (Fig. S6). Consistently, *Ca.* Omnitrophus, *Ca.* Nomurabacteria and *Pseudorhodobacter* have also been reported as prevalent taxa in waters from other cave and groundwater systems [42, 75–77], while *Polaromonas*, commonly found in polar and alpine environments, has been detected in aquatic cave habitats such as ice and cave pools [78, 79].

Although microorganisms can enter caves through both biotic (animal/human) and abiotic (air/water) agents [27, 28, 66, 80–82], our results do not indicate a strong transport connection between water and most cave substrates in Covadura. An exception may be represented by *Nitrospira*, which was detected at low abundances in cave air, but reached higher relative abundances in sediments, biofilms and several water samples, particularly in the inner cave sector. This pattern suggests that, for specific taxa, water may act as a preferential pathway for microbial distribution within the cave.

Overall, these results indicate that the Covadura waters function primarily as a distinct ecological habitat rather than as a general microbial reservoir or transport pathway for the cave microbiome, a pattern consistent with the arid to semiarid climate that usually prevails in the surrounding Sorbas karst region [45, 46].

### Cave air as a Key Dispersal Vector Linking External Inputs and Internal Reservoirs

Comparative analyses indicate that cave air, rather than water, acts as a preferential transport pathway for microbial dispersal in Covadura Cave. The cave aerobiome exhibited alpha-diversity levels comparable to those observed in sediments, rocks and biofilms, highlighting its ecological complexity. In contrast to water, cave air does not represent a stable habitat, but instead functions as a key dispersal medium linking surface soils with internal cave reservoirs.

Photosynthetic *Cyanobacteriota* were consistently detected in all air samples and in exterior soils, suggesting an external origin and airborne transport into the cave (Fig. 2). As cyanobacteria require light, they are typically restricted to illuminated cave galleries [1]; however, no natural or artificial light is present in the intermediate and inner sectors of Covadura Cave. Interestingly, *Cyanobacteriota* showed lower relative abundances in intermediate cave air samples (e.g. P2-A and P4-A) compared to both exterior air and air from more internal sectors. This pattern may reflect differences in the ventilation regime, as intermediate sectors are characterized by higher ventilation rates, whereas bioaerosols may accumulate more efficiently in less ventilated inner areas.

Several dominant taxa in soils above the cave, such as *Rubrobacter*, *Blastococcus*, AKIW781, WD2101_soil_group, RB41, and 0319-7L14 were frequently detected in air samples but showed only weak persistence within the cave. *Massilia*, *Hymenobacter*, and *Geodermatophilus* were also commonly recovered in air samples but were rare or absent on terrestrial cave substrates. Many of these genera have been considered components of the core microbiome of soils and airborne dust in arid environments, including the nearby Tabernas Desert [45, 46, 83–86]. Their lack of enrichment of these taxa on cave substrates supports their interpretation as passively transported airborne inputs rather than as lineages selectively adapted to subterranean conditions.

The high abundances of *Acinetobacter*, uncultured *Enterobacteriaceae* and *Sphingomonas* in the sample P4-A are noticeable (Fig. 5). As discussed above [28], this sampling site is a unique microbial hotspot shaped by specific environmental factors, such as a higher content of organic matter. Such localized conditions contrast with the general cave-air pattern observed for resident cave-associated taxa such as wb1-P19 and *Crossiella*, which were abundant in sediments, biofilms, and cave air, but present at very low abundances in exterior air and soils. Notably, *Crossiella*, wb1-P19 and members of the *Euzebyaceae* were also detected in air samples from another cave within the gypsum karst of Sorbas. In that cave, *Euzebyaceae* reached higher relative abundances than in Covadura, but based on the available data from both studies, it is not possible to define a clear role of air transport for this group [28].

Our results, for the first time, provide strong evidence that cave air acts as an important vector for both the introduction of external microorganisms and the internal redistribution of resident cave populations. This interpretation aligns with previous studies in a Paleolithic rock-art cave located in an Atlantic climate, where air circulation and resuspension of sediment particles were proposed as dispersal mechanisms for cave biofilms [42]. To our knowledge, only a limited number of studies have combined NGS-based analyses of cave air with multiple cave substrates [20, 48]. Zhu et al. [20] showed that cave air hosts a distinct microbial community, and Biagioli et al. [8] highlighted large-scale connections between cave systems and surface environments, but neither study explicitly addressed the role of air in the redistribution of core cave taxa. Nevertheless, the comparison of Covadura with other cave microbiomes supports the relevance of airborne transport under contrasting environmental settings.

## Conclusions and Future Directions

These findings establish a baseline ecological framework for understanding microbial dynamics in natural subterranean environments and underscore the ecological significance of these unique ecosystems. In Covadura Cave, biofilms, sediments, and waters act as relatively stable microbial reservoirs, whereas air functions as a selective vector that mediates both microbial entry and internal redistribution.

The limited role of water as a dispersal pathway observed in this study should be interpreted within the environmental context of Covadura Cave, located in a semi-arid region of southeastern Spain. In cave systems characterized by more humid climates or by active underground rivers or lakes, water may play a more prominent role not only as a habitat but also as a transport medium for microorganisms.

From a conservation and management perspective, our results highlight the importance of maintaining microclimatic stability and monitoring both aquatic and terrestrial microbial communities. Entrance and ecotone zones emerge as critical transitional buffers that help preserve internal cave stability. Consequently, environmental alterations in these transitional areas, such as shifts in humidity, temperature, or external inputs of organic matter, nutrients, or pollutants, are likely to exert very intense impacts on subterranean microbial consortia.

Together, this study emphasizes that habitat specialization and airborne dispersal structure cave microbiomes, reinforcing the need to integrate microbial ecology into the long-term conservation of subterranean ecosystems.

## Supplementary Information

Below is the link to the electronic supplementary material.

Supplementary Material 1 (DOCX 4.67 MB)

## Data Availability

The 16 S rRNA gene sequences and accompanying metadata from this study were deposited in the Sequence Read Archive (SRA) of NCBI under the project number PRJNA1237874.
