# Supplementary material for: Habitat Specialization and Airborne Dispersal Shape the Microbiome of a Gypsum Karst Cave
Source: Microb Ecol. 2026 Mar 20;89:88. doi: 10.1007/s00248-026-02745-y (PMC13046682; doi:10.1007/s00248-026-02745-y)
Supplement: Supplementary file 1 [file 248_2026_2745_MOESM1_ESM.docx]

**SUPPLEMENTARY INFORMATION**

# **Habitat Specialization and Airborne Dispersal Shape the Microbiome of a Gypsum Karst Cave**

Tamara Martin-Pozas^1^, Angel Fernandez-Cortes^2^, Jose Maria Calaforra^2^, Guillermo Ledesma-Hernández^3^, Soledad Cuezva^3^, Sergio Sanchez-Moral^3^, Cesareo Saiz-Jimenez^1^, Valme Jurado^1^

^1^Instituto de Recursos Naturales y Agrobiologia de Sevilla, IRNAS-CSIC, 41012 Sevilla, Spain. tmpozas@csic.es; v.jurado@csic.es; saiz@irnase.csic.es

^2^Departamento de Biologia y Geologia, Universidad de Almeria, Almeria, Spain. acortes@ual.es; jmcalaforra@ual.es

^3^Museo Nacional de Ciencias Naturales, MNCN-CSIC, Madrid. Spain. guillermo.ledesma@mncn.csic.es, scuezva@csic.es, ssmilk@mncn.csic.es

*Corresponding author: acortes@ual.es

**Environmental and physicochemical characterization**

*Microscopy*

In Covadura Cave, it is difficult to distinguish the types of biofilms with naked eye. They are complex and diverse, and sometimes they overlap at the same location. To correctly classify them, we first analyzed them in the lab using a Leica M165C stereoscopic microscope, and then under a FEI INSPECT environmental scanning electron microscope (ESEM). For ESEM, samples were stored in a silica-gel desiccator for one week to eliminate excess water and coated with a thin conductive layer of gold particles to improve secondary electron emission, image contrast and resolution. The biofilms were classified according to the following characteristics: color, size, elevation, shape, margin, and degree of mineralization.

*Environmental monitoring*

Cave air monitoring was carried out to gather essential data for assessing the current environmental conditions within the cave coeval to the field campaigns for microbiological sampling (June 28, 2022 and October 5, 2023). This included measurements of air temperature, relative humidity, and the gaseous composition of the cave air, as well as samples from the external soil and the local background atmosphere.

Air temperature and relative humidity were measured along the points of the microbiology sampling network in Figure 1, using a handheld XP200 monitor (Lufft, Germany). This device featured an external PT100 1/10 DINB probe for temperature, with an accuracy of ±[0.03+0.002∗measurement], and a capacity probe for relative humidity, which operates within a measurement range of 0-100% and has an accuracy of ±3% for readings above 90%.

In addition, air samples were gathered using a portable air compressor operating at 0.4 L·min⁻¹ and stored in 1-L RITTER gas bags. Air samples were analysed within 48 hours for CO₂ and CH₄ molar fractions, as well as δ¹³C-CO₂ and δ¹³C-CH₄ values, utilizing a G2201-i CRDS analyser from Picarro, USA.

*Physicochemical Sediment Analyses*

Sediment samples were air-dried, crushed, sieved (2 mm), and ground (< 60 µm) to determine total organic carbon (TOC), total nitrogen (TN), and metal/trace element concentrations. The pH and electrical conductivity (EC) of the sediments were measured in a 1:2.5 sample/H_2_O extract, and in a 1:5 sample/H_2_O, respectively, using a CRISON MultiMeter MM 41 after 1 hour of shaking. TOC and TN were analyzed with a Primacs SNC 100 IC-E Skalar elemental analyzer; CO₂ and N₂ were detected via infrared and thermal conductivity detectors, respectively. Organic carbon was calculated as the difference between total carbon and inorganic carbon (IC), after acidification of the sample.

Available phosphorus was extracted with 0.5 M NaHCO₃ (pH 8.5) following the method of Olsen and Sommers [1], and measured colorimetrically using a Bran+Luebbe AutoAnalyzer AA III. Ammonium and nitrate were extracted with 1 M KCl and analyzed using the same analyzer [2]. Ca, Mg, K, and Na were extracted using 1 M ammonium acetate (pH 7) and analyzed via inductively coupled plasma optical emission spectrometry (ICP-OES) in a VARIAN ICP 720-ES). For metal and trace element analysis, samples were digested with aqua regia (HNO₃:HCl, 1:3) in a microwave digester. Resulting concentrations were measured in the VARIAN ICP 720-ES.

*Geochemical water analysis*

Water, pH and EC were measured directly using a CRISON MultiMeter MM 41. Ammonium, nitrate, nitrite, and chloride were quantified using a Bran+Luebbe AutoAnalyzer with spectrophotometric detection. Alkalinity was measured by titration with 0.02 N H₂SO₄ using a methyl red/bromocresol green indicator mix. Total carbon and nitrogen were analyzed with a Shimadzu TOC-VCSH analyzer. Metals were quantified by ICP-OES after filtration (0.45 µm nylon filters) and acidification with 2% (v/v) HNO_3_ (30%).

**Supplementary Table S1**. Sample description

| **Site-sample code** | **Type**^2^ | **Date** | **Location**^1^ | **Microenvironmental zoning**^2^ | **Distance to exterior (m)** |
| --- | --- | --- | --- | --- | --- |
| **P0-S (1)** | Exterior soil | 2023 | Exterior (P0) | Exterior | 0 |
| **P0-S (2)** | Exterior soil | 2023 | Exterior (P0) | Exterior | 0 |
| **P0-A (1)** | Exterior air | 2023 | Exterior (P0) | Exterior | 0 |
| **P0-A (2)** | Exterior air | 2023 | Exterior (P0) | Exterior | 0 |
| **P2-A** | Cave air | 2023 | Cave (P2) | Ecotone | 33 |
| **P4-A** | Cave air | 2023 | Cave (P4) | Intermediate | 73 |
| **P6/7-A** | Cave air | 2023 | Cave (P6/7) | Isolated | 176 |
| **P9-A** | Cave air | 2023 | Cave (P9) | Isolated | 236 |
| **P1-S** | Cave Sediment | 2022 | Cave (P1) | Ecotone | 12 |
| **P3-S** | Cave Sediment | 2022 | Cave (P3) | Ecotone | 57 |
| **P4-S** | Cave Sediment (Marls) | 2022 | Cave (P4) | Intermediate | 73 |
| **P6-S** | Cave Sediment | 2022 | Cave (P6) | Intermediate | 153 |
| **P9-S** | Cave Sediment | 2022 | Cave (P9) | Isolated | 236 |
| **P4-By** | Yellow biofilm | 2022 | Cave (P4) | Intermediate | 73 |
| **P10-By** | Yellow biofilm | 2010 | Cave (P10) | Intermediate | 70 |
| **P6-By (1)** | Yellow biofilm | 2022 | Cave (P6) | Intermediate | 153 |
| **P6-By (2)** | Yellow biofilm | 2022 | Cave (P6) | Intermediate | 153 |
| **P6-By (3)** | Yellow biofilm | 2010 | Cave (P6) | Intermediate | 153 |
| **P8-By** | Yellow biofilm | 2010 | Cave (P8) | Isolated | 236 |
| **P4-Bw (1)** | White biofilm | 2010 | Cave (P4) | Intermediate | 73 |
| **P4-Bw (2)** | White biofilm | 2022 | Cave (P4) | Intermediate | 73 |
| **P10-Bw** | White biofilm | 2010 | Cave (P10) | Intermediate | 70 |
| **P6-Bw** | White biofilm | 2022 | Cave (P6) | Intermediate | 153 |
| **P8-Bw** | White biofilm | 2022 | Cave (P8) | Isolated | 236 |
| **P4-W** | Cave water pool | 2023 | Cave (P4) | Intermediate | 73 |
| **P6/7-W** | Cave drip water | 2023 | Cave (P6/7) | Isolated | 176 |
| **P8-W** | Cave water pool | 2023 | Cave (P8) | Isolated | 236 |
| **P9-W** | Cave water pool | 2023 | Cave (P9) | Isolated | 256 |

^1^ For sample locations, see also Figure 1.

^2^ For ecological zone description, see also Supplementary Table S2.

**Supplementary Table S2**. Covadura Cave environmental parameters

| **Microenvironmental zoning** ^1^ | **Site-sample code** | **Type of sample** | **Date** | **Distance to exterior (m)** | **T (^o^ C)** | **HR (%)** | **CO_2_ (ppm)** | **CH_4_ (ppm)** |
| --- | --- | --- | --- | --- | --- | --- | --- | --- |
| Exterior (P0) | P0-S (1) | Exterior soil | Jun 2022 – Oct 2023 | 0 | 29.0 ± 0.87 | 41.4 ± 2.43 | 426.0 ± 1.15 | 2.00 ± 0.00 |
| Exterior (P0) | P0-S (2) | Exterior soil | Jun 2022 – Oct 2023 | 0 | 29.0 ± 0.87 | 41.4 ± 2.43 | 426.0 ± 1.15 | 2.00 ± 0.00 |
| Exterior (P0) | P0-A (1) | Exterior air | Jun 2022 – Oct 2023 | 0 | 29.0 ± 0.87 | 41.4 ± 2.43 | 426.0 ± 1.15 | 2.00 ± 0.00 |
| Exterior (P0) | P0-A (2) | Exterior air | Jun 2022 – Oct 2023 | 0 | 29.0 ± 0.87 | 41.4 ± 2.43 | 426.0 ± 1.15 | 2.00 ± 0.00 |
| Cave/Ecotone (P1) | P1-S | Cave sediment | Jun 2022 – Oct 2023 | 12 | 19.5 ± 0.12 | 84.2 ± 3.03 | 458.5 ± 15.9 | 1.94 ± 0.00 |
| Cave/Ecotone (P2) | P2-A | Cave air | Jun 2022 – Oct 2023 | 33 | 17.0 ± 0.58 | 89.5 ± 3.17 | 482.5 ± 8.37 | 1.95 ± 0.00 |
| Cave/Ecotone (P3) | P3-S | Cave sediment | Jun 2022 – Oct 2023 | 57 | 15.8 ± 0.29 | 91.4 ± 1.36 | 450.0 ± 5.77 | 1.96 ± 0.01 |
| Cave/Intermediate (P10) | P10-Bw | White biofilm | Oct 2010 – Nov 2010 | 70 | NA | NA | NA | NA |
| Cave/Intermediate (P10) | P10-By | Yellow biofilm | Jun 2022 – Oct 2023 | 70 | NA | NA | NA | NA |
| Cave/Intermediate (P4) | P4-A | Cave air | Jun 2022 – Oct 2023 | 73 | 13.9 ± 0.12 | 92.0 ± 0.75 | 460.0 ± 4.04 | 1.79 ± 0.00 |
| Cave/Intermediate (P4) | P4-Bw (1) | White biofilm | Jun 2022 – Oct 2023 | 73 | 13.9 ± 0.12 | 92.0 ± 0.75 | 460.0 ± 4.04 | 1.79 ± 0.00 |
| Cave/Intermediate (P4) | P4-Bw (2) | White biofilm | Jun 2022 – Oct 2023 | 73 | 13.9 ± 0.12 | 92.0 ± 0.75 | 460.0 ± 4.04 | 1.79 ± 0.00 |
| Cave/Intermediate (P4) | P4-By | Yellow biofilm | Jun 2022 – Oct 2023 | 73 | 13.9 ± 0.12 | 92.0 ± 0.75 | 460.0 ± 4.04 | 1.79 ± 0.00 |
| Cave/Intermediate (P4) | P4-S | Cave sediment | Jun 2022 – Oct 2023 | 73 | 13.9 ± 0.12 | 92.0 ± 0.75 | 460.0 ± 4.04 | 1.79 ± 0.00 |
| Cave/Intermediate (P4) | P4-W | Cave water | Jun 2022 – Oct 2023 | 73 | 13.9 ± 0.12 | 92.0 ± 0.75 | 460.0 ± 4.04 | 1.79 ± 0.00 |
| Cave/Intermediate (P5) | NA | NA | Jun 2022 – Oct 2023 | 118 | 13.45 ± 0.14 | 93.5 ± 0.41 | 458.5 ± 2.02 | 1.78 ± 0.00 |
| Cave/Intermediate (P6) | P6-Bw | White biofilm | Jun 2022 – Oct 2023 | 153 | 12.45 ± 0.38 | 93.05 ± 0.26 | 453.5 ± 5.48 | 1.62 ± 0.05 |
| Cave/Intermediate (P6) | P6-By (1) | Yellow biofilm | Jun 2022 – Oct 2023 | 153 | 12.45 ± 0.38 | 93.05 ± 0.26 | 453.5 ± 5.48 | 1.62 ± 0.05 |
| Cave/Intermediate (P6) | P6-By (2) | Yellow biofilm | Jun 2022 – Oct 2023 | 153 | 12.45 ± 0.38 | 93.05 ± 0.26 | 453.5 ± 5.48 | 1.62 ± 0.05 |
| Cave/Intermediate (P6) | P6-By (3) | Yellow biofilm | Jun 2022 – Oct 2023 | 153 | 12.45 ± 0.38 | 93.05 ± 0.26 | 453.5 ± 5.48 | 1.62 ± 0.05 |
| Cave/Intermediate (P6) | P6-S | Cave sediment | Jun 2022 – Oct 2023 | 153 | 12.45 ± 0.38 | 93.05 ± 0.26 | 453.5 ± 5.48 | 1.62 ± 0.05 |
| Cave/Intermediate (P6) | P6-Bw | White biofilm | Jun 2022 – Oct 2023 | 153 | 12.1 ± 0.29 | 92.8 ± 0.35 | 465.5 ± 7.79 | 1.51 ± 0.03 |
| Cave/ Isolated (P6/7) | P6/7-A | Cave air | Jun 2022 – Oct 2023 | 176 | 12.1 ± 0.29 | 92.8 ± 0.35 | 465.5 ± 7.79 | 1.51 ± 0.03 |
| Cave/ Isolated (P6/7) | P6/7-W | Cave water | Jun 2022 – Oct 2023 | 176 | 12.1 ± 0.29 | 92.8 ± 0.35 | 465.5 ± 7.79 | 1.51 ± 0.03 |
| Cave/Isolated (P7) | NA | NA | Jun 2022 – Oct 2023 | 197 | 11.75 ± 0.20 | 92.6 ± 0.40 | 477.5 ± 10.1 | 1.15 ± 0.05 |
| Cave/Isolated (P8) | P8-Bw | White biofilm | Jun 2022 – Oct 2023 | 236 | 11.45 ± 0.20 | 92.85 ± 0.84 | 489.5 ± 3.17 | 1.15 ± 0.05 |
| Cave/Isolated (P8) | P8-By | Yellow biofilm | Jun 2022 – Oct 2023 | 236 | 11.45 ± 0.20 | 92.85 ± 0.84 | 489.5 ± 3.17 | 1.15 ± 0.05 |
| Cave/Isolated (P9) | P9-A | Cave air | Jun 2022 – Oct 2023 | 256 | 11.8 ± 0.17 | 92.0 ± 1.80 | 513 ± 4.62 | 1.15 ± 0.03 |
| Cave/Isolated (P9) | P9-S | Cave sediment | Jun 2022 – Oct 2023 | 256 | 11.8 ± 0.17 | 92.0 ± 1.80 | 513 ± 4.62 | 1.15 ± 0.03 |
| Cave/Isolated (P9) | P9-W | Cave water | Jun 2022 – Oct 2023 | 256 | 11.8 ± 0.17 | 92.0 ± 1.80 | 513 ± 4.62 | 1.15 ± 0.03 |

**Supplementary Table S3**. Geochemical analysis of water samples

| **Water samples** | **pH** | **CE at 25°C [dS/m]** | **Organic C**  **[mg/l]** | **Inorganic C**  **[mg/l]** | **HCO_3_^-^**  **[mg/l]** | **NO_3_^-^**  **[mg/l]** | **Cl**  **[mg/l]** | **S [mg/k]** | **Na [mg/k]** | **K [mg/k]** | **Ca [mg/k]** | **Mg [mg/k]** |
| --- | --- | --- | --- | --- | --- | --- | --- | --- | --- | --- | --- | --- |
| **P4-W** | 7.59 | 2.30 | 6.1 | 10.0 | 292 | 3.6 | 38.9 | 590.2 | 23.3 | 3.3 | 598.1 | 17.7 |
| **P6/7-W** | 7.50 | 2.24 | 6.0 | 11.3 | 292 | 3.4 | 56.8 | 629.1 | 38.0 | 3.2 | 576.8 | 14.0 |
| **P8-W** | 7.58 | 2.25 | 6.2 | 10.3 | 205 | 5.2 | 65.8 | 568.7 | 34.0 | 2.8 | 580.1 | 10.8 |
| **P9-W** | 7.53 | 2.32 | 6.8 | 9.3 | 205 | 14.4 | 50.7 | 535.5 | 32.4 | 7.7 | 589.6 | 12.9 |

**Supplementary Table S4**. Geochemical analysis of sediment and soil samples

| **Sample** | **Type** | **pH** | **EC (mS/cm)** | **CaCO_3_ (%)** | **Organic C (%)** | **Organic Matter (%)** | **N Kjeldahl (%)** | **NO_3_^–^ (mg/kg)** | **NH₄⁺ (mg/kg)** | **Phosphorous (mg/kg)** | **Potassium**  **(mg/kg)** | **Calcium (mg/kg)** | **Magnesium (mg/kg)** |
| --- | --- | --- | --- | --- | --- | --- | --- | --- | --- | --- | --- | --- | --- |
| **P0-S** | Exterior Soil | 8.10 | 1.83 | 49.3 | 1.04 | 1.79 | NA | 1.40 | 2.00 | 1.40 | 132 | 10400 | 42.1 |
| **P1-S** | Cave Sediment | 7.73 | 2.78 | 31.0 | 0.28 | 0.48 | 0.044 | 79.8 | 2.20 | 15.4 | 1098 | 40698 | 331 |
| **P3-S** | Cave Sediment | 7.68 | 2.80 | 26.2 | 0.25 | 0.43 | 0.043 | 28.4 | 2.30 | 2.40 | 341 | 52889 | 365 |
| **P4-S** | Cave Sediment (Marl) | 7.81 | 1.46 | 63.6 | 1.15 | 1.99 | 0.026 | 2.50 | <0.1 | 2.20 | 65.6 | 8311 | 60.3 |
| **P6-S** | Cave Sediment | 7.61 | 2.52 | 5.10 | 0.06 | 0.30 | 0.037 | 3.56 | 2.20 | 5.30 | 379 | 9731 | 453 |
| **P9-S** | Cave Sediment | 7.78 | 2.18 | 57.3 | 0.39 | 0.67 | 0.027 | 2.70 | 2.10 | 5.60 | 12.6 | 31460 | 47.6 |

**Supplementary Table S5**. Covadura Cave biofilm classification

| **Sample** | **Biofilm morphology under Optical Microscope** | | | | | **Cell morphology under ESEM** | **Type** |
| --- | --- | --- | --- | --- | --- | --- | --- |
|  | **Color** | **Size*** | **Elevation** | **Shape** | **Margin** |  |  |
| **P4-By** | Yellow | 1.2.3.4 | Raised | Filamentous | Irregular | **-Long fluffy bacillary filaments (4 μm length. 0.6 μm width)**  **-Smooth cocci (0.3-1.5 µm)**  -Cocci with surface appendages (1-1.5 µm)  -Long bacillary (2 μm length. 1 μm width) filaments with surface appendages  -Long smooth filaments (0.1-0.2 μm width)  -Long bacillary smooth filaments (4 μm length. 0.6 μm width)  -Smooth cocci (0.2-0.3 µm) | Yellow biofilm |
| **P10-By** | Yellow | 1.2.3.4 | Raised | Filamentous | Irregular | **-Long fluffy bacillary filaments (4 μm length. 0.6 μm width) ****  **-Smooth cocci (0.3-1.5 µm) ****  -Long bacillary smooth filaments (4 μm length. 0.6 μm width) | Yellow biofilm |
| **P6-By** | Yellow | 1.2.3.4 | Raised | Filamentous | Irregular | **-Long fluffy bacillary filaments (4 μm length. 0.6 μm width) ****  **-Smooth cocci (0.3-1.5 µm) ****  -Cocci with surface appendages (1-1.5 µm) | Yellow biofilm |
| **P8-By** | Yellow | 1.2.3.4 | Raised | Filamentous | Irregular | **-Long fluffy bacillary filaments (4 μm length. 0.6 μm width) ****  **-Smooth cocci (0.3-1.5 µm) ****  -Cocci with surface appendages (1-1.5 µm)  -Long bacillary (2 μm length. 1 μm width) filaments with surface appendages  -Long smooth filaments (0.1-0.2 μm width)  -Long bacillary smooth filaments (4 μm length. 0.6 μm width)  -Smooth cocci (0.2-0.3 µm) | Yellow biofilm |
| **P4-Bw** | White | 2.3.4 | Raised | Filamentous | Irregular | -Cocci with surface appendages (1-1.5 µm)  -Long bacillary (2 μm length. 0.6 μm width) filaments with surface appendages  -Long smooth filaments (0.1-0.2 μm width)  -Long bacillary (2 μm length. 1 μm width) filaments with surface appendages | White biofilm |
| **P10-Bw** | White | 1.2.3 | Raised | Filamentous | Irregular | -Long smooth filaments (0.2-0.3 μm width)  -Smooth cocci (0.2-0.3 µm) | White biofilm |
| **P6-Bw** | White | 1.2.3 | Raised | Filamentous | Irregular | -Long smooth filaments (0.2-0.3 μm width) | White biofilm |
| **P8-Bw** | White | 1.2.3 | Raised | Filamentous | Irregular | -Long bacillary (2 μm length. 1 μm width) filaments with surface appendages | White biofilm |

*1. Punctiform (<0.5mm). 2. Small (<1mm). 3. Medium (1mm). 4. Large (>1mm)

*Most abundant cellular morphologies

**Supplementary Table S6**. Chao1, Shannon, and Simpson’s indexes

| **SAMPLES** | **RICHNESS** | | **DIVERSITY** | |
| --- | --- | --- | --- | --- |
|  | **Observed** | **Chao1** | **Shannon** | **Simpson** |
| P0-S(1) | 2990 | 3015.13 | 7.35 | 1.00 |
| P0-S(2) | 3484 | 3537.28 | 7.40 | 1.00 |
| P0-A (1) | 1972 | 1981.26 | 6.90 | 1.00 |
| P0-A (2) | 1816 | 1826.55 | 6.73 | 1.00 |
| P2-A | 3057 | 3078.40 | 7.38 | 1.00 |
| P4-A | 1466 | 1517.03 | 4.94 | 0.96 |
| P6/7-A | 3060 | 3091.57 | 7.30 | 1.00 |
| P9-A | 2527 | 2544.24 | 7.03 | 1.00 |
| P1-S | 980 | 980.86 | 5.84 | 0.99 |
| P3-S | 617 | 618.00 | 4.87 | 0.97 |
| P4-S | 1083 | 1090.39 | 5.19 | 0.97 |
| P6-S | 501 | 501.50 | 5.18 | 0.99 |
| P9-S | 1424 | 1424.00 | 6.82 | 1.00 |
| P4-By | 554 | 558.13 | 3.53 | 0.81 |
| P10-By | 640 | 641.11 | 4.52 | 0.95 |
| P6-By(1) | 611 | 611.63 | 4.16 | 0.92 |
| P6-By(2) | 241 | 241.00 | 2.39 | 0.66 |
| P6-By(3) | 928 | 931.90 | 5.37 | 0.98 |
| P8-By | 335 | 337.50 | 2.99 | 0.77 |
| P4-Bw(1) | 411 | 411.00 | 3.09 | 0.73 |
| P4-Bw(2) | 421 | 424.50 | 3.73 | 0.93 |
| P10-Bw | 739 | 739.20 | 3.75 | 0.76 |
| P6-Bw | 662 | 666.11 | 4.56 | 0.93 |
| P8-Bw(1) | 477 | 479.50 | 3.51 | 0.78 |
| P4-W | 2880 | 2899.68 | 7.53 | 1.00 |
| P6/7-W | 3142 | 3170.39 | 7.49 | 1.00 |
| P8-W | 1933 | 1972.50 | 5.57 | 0.95 |
| P9-W | 1963 | 2068.34 | 4.84 | 0.92 |

**Supplementary Table S7**. ANOSIM and PERMANOVA analysis

| **ANOSIM (Analysis of Similarities)** | **Sample size** | **Permutations** | **R** | **P** |  |
| --- | --- | --- | --- | --- | --- |
| **Differences between exterior and cave environments** |  |  |  |  |  |
| Cave Sediment (N5) vs Exterior soil (N2) | **7** | **999** | **0.7273** | **0.041** |  |
| Cave air (N4) vs Exterior air (N2) | 6 | 719 | 0.7143 | 0.0667 |  |
| **Within-Cave habitats (Air, Biofilm, Sediment, Water)** | **24** | **999** | **0.8173** | **0.0001** |  |
| **Pairwise** | **Sample size** | **Permutations** | **R** | **P** |  |
| Cave air (N4) vs Cave biofilm (N11) | **15** | **999** | **0.9419** | **0.0009** |  |
| Cave air (N4) vs Cave sediment (N5) | **9** | **999** | **0.3188** | **0.0317** |  |
| Cave air (N4) vs Cave water (N4) | **8** | **999** | **1.000** | **0.0263** |  |
| Biofilm (N11) vs Cave sediment (N5) | **16** | **999** | **0.7337** | **0.0005** |  |
| Biofilm (N11) vs Cave water (N4) | **15** | **999** | **0.9940** | **0.0009** |  |
| Cave sediment (N5) vs Cave water (N4) | **9** | **999** | **0.4500** | **0.0402** |  |
| **Within-Cave biofilm types (Yellow (N6), White (N5))** | **11** | **999** | **0.3045** | **0.0301** |  |
| **Biofilms collected in 2010 (N5) vs 2022 (N6)** | **11** | **999** | **0.2747** | **0.0440** |  |
| **PERMANOVA** | **Sample size** | **Permutations** | **Pseudo-F** | **R2** | **P** |
| **Differences between exterior and cave environments** |  |  |  |  |  |
| Cave sediment (N5) vs Exterior soil (N2) | **7** | **999** | **2.0757** | **0.050** | **0.0003** |
| Cave air (N4) vs Exterior air (N2) | 6 | 719 | 1.5181 | 0.2751 | 0.0667 |
| **Within-Cave habitats (Air, Biofilm, Sediment, Water)** | **24** | **999** | **2.7005** | **0.27335** | **0.0001** |
| **Within-Cave habitats (****Microenvironmental zoning)** | 24 | 999 | 1.1379 | 0.07678 | 0.1838 |
| **Pairwise** | **Sample size** | **Permutations** | **Pseudo-F** | **R2** | **P** |
| Cave air (N4) vs Cave biofilm (N11) | **15** | **999** | **4.0097** | **0.2357** | **0.0011** |
| Cave air (N4) vs Cave sediment (N5) | **9** | **999** | **2.3650** | **0.2525** | **0.0079** |
| Cave air (N4) vs Cave water (N4) | **8** | **999** | **4.1428** | **0.4084** | **0.0321** |
| Biofilm (N11) vs Cave sediment (N5) | **16** | **999** | **2.1753** | **0.1345** | **0.0001** |
| Biofilm (N11) vs Cave water (N4) | **15** | **999** | **3.9804** | **0.2344** | **0.0007** |
| Cave sediment (N5) vs Cave water (N4) | **9** | **999** | **2.1448** | **0.2345** | **0.0079** |
| **Within-Cave biofilm types (Yellow (N6), White (N5))** | **11** | **999** | **1.5146** | **0.1440** | **0.0377** |
| Biofilms collected in 2010 (N5) vs 2022 (N6) | 11 | 999 | 1.8422 | 0.1699 | 0.1530 |

**Supplementary Figure S1.** Images of yellow biofilms from Covadura Cave. (a) Sampling point 4; (b) sampling point 10; (c) sampling point P6; and (d) sampling point 8. See Figure 1 for the map of the cave showing the sampling points.

**Supplementary Figure S2**. Images of white biofilms from Covadura Cave. (a) Sampling point 4; (b) sampling point 10; (c) sampling point P6; and (d) sampling point 8. See Figure 1 for the map of the cave showing the sampling points.

**Supplementary Figure S3**. Representative cellular structures observed in yellow biofilms. (a) General view of the biofilm under optical microscopy. (b) Overview of the biofilm surface under scanning electron microscopy. (c. d) Detailed views of predominant cell morphologies within the yellow biofilm: elongated bacillary filaments with a fluffy appearance and smooth cocci.

**Supplementary Figure S4**. White biofilms. (a) General view of the biofilm under optical microscopy. (b) Overview of the biofilm surface under scanning electron microscopy. (c-f) Detailed views of main cell structures within the white biofilms: (c-d) long bacillary filaments with ornamented surfaces. (f) clustered cocci with ornamented surfaces.

**Supplementary Figure S5**. Rarefaction curves

**Supplementary Figure S6**. Indicator taxa for different cave and exterior environments (IndVal analysis). Bubble plot representing the averaged relative abundances of indicator genera associated with different sample types. Color intensity reflects the corresponding q-value of the IndVal test. Only the most abundant taxa with sufficient statistical support are included (IndVal index > 0.6 and q-value < 0.05). Colored indicator values and indicator groups shown in the left panel correspond to the different indicator groups.

**Supplementary Figure S7**. Microbial co-occurrence network in cave sediments. Nodes represent bacterial genera detected in cave sediments and node size is proportional to mean relative abundance. Only most abundant taxa are colored by phylum, while low-abundance phyla are grouped as Other. Labels and black outlines indicate the most abundant genera and highly connected hub taxa (based on degree centrality). Edges represent significant pairwise associations between genera. Red and blue edges indicate positive and negative correlations, respectively.

**Supplementary Figure S8**. Co-occurrence network in yellow biofilms. Nodes represent bacterial genera detected in yellow biofilms and node size is proportional to mean relative abundance. Only most abundant taxa are colored by phylum, while low-abundance phyla are grouped as Other. Labels and black outlines indicate the most abundant genera and highly connected hub taxa (based on degree centrality) Edges represent significant pairwise associations between genera, with red and blue edges indicating positive and negative correlations.

**Supplementary Figure S9**. Bacterial co-occurrence network in white biofilms. Nodes represent bacterial genera detected in white biofilms and node size is proportional to mean relative abundance. Only most abundant taxa are colored by phylum, while low-abundance phyla are grouped as Other. Labels and black outlines indicate the most abundant genera and highly connected hub taxa (based on degree centrality) Edges represent significant pairwise associations between genera, with red and blue edges indicating positive and negative correlations.

**References**

1. Olsen SR, Sommers LE (1982) Phosphorus. In: Page AL, Miller RH, Deeney DR (eds) Methods of Soil Analysis. Part 2. ASA, Madison, Wisconsin, USA. pp. 403–430. https://doi.org/10.2134/agronmonogr9.2.2ed
2. Kachurina OM, Zhang, H, Raun WR et al (2000) Simultaneous determination of soil aluminum. ammonium- and nitrate-nitrogen using 1 *M* potassium chloride extraction. Comm Soil Sci Plant Anal 31:893–903. https://doi.org/10.1080/00103620009370485
